# Managing Multifactorial Deep Vein Thrombosis in an Adolescent: A Complex Case Report

**DOI:** 10.2478/jccm-2024-0024

**Published:** 2024-07-31

**Authors:** Măriuca Mănescu, Alina Grama, Andreea Ligia Dincă, Mihaela Chinceșan

**Affiliations:** Emergency County Hospital Targu-Mures, Targu Mures, Romania; George Emil Palade University of Medicine, Pharmacy, Science, and Technology of Targu Mures, Romania

**Keywords:** deep vein thrombosis, DVT, high-risk, pediatric patient, risk factor

## Abstract

**Introduction:**

Although rarely diagnosed in the pediatric population, deep vein thrombosis (DVT) is experiencing a growing incidence, while continuously acquiring different nuances due to the widening range of risk factors and lifestyle changes in children and adolescents.

**Case presentation:**

A 17-year-old female within four weeks after child delivery was admitted to our clinic due to a six-month history of pain in the left hypochondriac region. After a thorough evaluation, the presence of a benign splenic cyst was revealed, which was later surgically removed. Following the intervention, the patient developed secondary thrombocytosis and bloodstream infection which, together with pre-existing risk factors (obesity, compressive effect of a large cyst, the postpartum period, the presence of a central venous catheter, recent surgery, and post-operative mobilization difficulties) led to the occurrence of extensive DVT, despite anticoagulant prophylaxis and therapy with low-molecular-weight heparin.

**Conclusions:**

DVT raises many challenges for the pediatrician, requiring a personalized approach. Although rare, pediatric patients with multiple concomitant high-risk factors should benefit from interdisciplinary care as DVT may not respond to standard therapy in such cases and rapidly become critical. Continual efforts to better understand and treat this condition will contribute to improved outcomes for pediatric patients affected by DVT.

## Introduction

While uncommon in young people, there is growing concern about the increasing prevalence of deep vein thrombosis (DVT) in children and adolescents [1]. This is presumably linked to changes in modern lifestyle habits [2]. As the medical landscape evolves, the risk factors of DVT may become more diverse, potentially leading to more complex cases that require careful management. Continual research is essential for comprehending DVT and improving therapeutic options.

Written informed consent was obtained from the parents for the publication of this case report.

## Case Presentation

### Clinical presentation and initial investigations

A 17-year-old female (body mass index of 31 kg/m^2^, that is above the 96^th^ percentile) was admitted with pain in the left hypochondriac region for the past six months. She had given birth approximately one month before admission. The clinical examination revealed pain in the left hypochondrium and hypogastrium, radiating to the left shoulder. Hepatomegaly and splenomegaly were also observed. Laboratory tests on admission revealed no notable findings, except for an an erythrocyte sedimentation rate of 45 mm/h.

The abdominal ultrasound showed a large splenic cystic mass (123.9×109.9 mm) with well-defined borders, no Doppler signal, and normal echogenicity. Subsequently, a computed tomography (CT)-scan was conducted, confirming a sizeable splenic mass pressuring the pancreas tail and left kidney. This finding prompted several other investigations which all yielded results within the normal range: tumor markers (AFP, CA-125, β-hCG), anti-Echinococcus antibodies, serological tests for hepatitis B and C, HIV and TORCH infections, chest X-ray, EEG, and a neurological examination.

### Diagnosis

Splenic cysts (primary or secondary) are a relatively rare finding. Primary splenic cysts can be either parasitic or nonparasitic, while secondary splenic cysts are usually the result of abdominal trauma [5]. Establishing the most likely etiology is important given that parasitic and nonparasitic cysts require different therapeutical approaches [5]. In central Europe, the nonparasitic etiology is more frequent [5]. Primary splenic cysts can also mimic malignant tumors or metastases [6]. Considering patient history and negative laboratory results, it was concluded that a nonparasitic primary splenic cyst was the most likely diagnosis.

### Initial management and prognosis

Based on the size and observed compressive effect, the decision was made to proceed with surgical removal. As part of the pre-operative routine, a central venous catheter (CVC) was inserted in the right femoral vein. Splenectomy was performed and the patient was expected to have a favorable recovery. Post-surgery, among other treatments, the patient was given preventive anticoagulants with subcutaneous injections of Enoxaparin sodium 40 mg QD (low molecular weight heparin, LMWH). While sequential compression devices would have been an optimal choice for mechanical prophylaxis given the patient’s increased risk of DVT, they were not available in our clinic. The histopathological examination identified the formation as a benign mesothelial splenic cyst.

### Case progression and outcome

The patient was mobilized on the 5th day after the surgery and the clinical and paraclinical progress was generally positive until the 8th day, when the patient started experiencing fever (38.6°C), pain in the right lower limb, difficulty in mobilization, and reported pain and discomfort at the site of the CVC. Subsequently, the CVC was removed and a CVC tip culture revealed the presence of *Staphylococcus hominis*. According to the laboratory’s antimicrobial susceptibility testing report, Clindamycin therapy was initiated, but no improvements were noticed. A postoperative CT-scan showed a fluid collection within the splenic chamber, indicating a possible splenic abscess. Multiple interdisciplinary consultations were conducted, and the antibiotic therapy was adjusted.

The right lower limb pain raised the suspicion of a potential postoperative complication: deep vein thrombosis. Post-splenectomy monitoring also revealed thrombocytosis, with platelet counts eventually reaching levels as high as 1,067×10^3^/μL, and a positive D-dimer test (1249 ng/mL). Further, a Doppler ultrasound was conducted, which revealed thrombi in the external iliac, common femoral, and femoral right veins. Consequently, the LMWH anticoagulant therapy was adjusted from a prophylactic to a therapeutic dose (Enoxaparin sodium 80 mg BID by subcutaneous injection), and hydration infusions were administered. Notably, the therapeutic administration of LMWH was not monitored due to the unavailability of the anti-Xa assay in our hospital’s laboratory.

It is important to note that there was no documented family history of venous thrombotic events. The results of the screening test for inherited thrombophilia, including antithrombin III, protein C, protein S levels, factor V Leiden mutation, prothrombin mutation (factor II), and MTHFR gene mutations, were all negative.

Despite the change of LMWH regimen, the patient’s clinical condition further deteriorated. Subsequent ultrasound examinations indicated the extension of thrombi to the inferior vena cava and also to the left lower limb (common iliac, common femoral, and femoral veins).

After the completion of antibiotic therapy and remission of the infectious process, the patient’s fever and inflammatory markers subsided. Nevertheless, despite complete recanalization in the inferior vena cava and partial recanalization in the other affected veins, the patient still experienced pain in the lower limb. During the physical examination, no local changes suggestive of a post-thrombotic syndrome were observed, such as pretibial edema, redness, skin induration, hyperpigmentation, or venous ulcers. A neuropsychiatric evaluation revealed a mixed anxiety-depressive disorder (possibly postpartum-related) and anxiolytic therapy led to gradual improvements.

## Discussion

DVT, traditionally considered a concern in adult medicine, is now increasingly recognized in pediatrics, with a rising incidence among children and adolescents and serious potential consequences such as post-thrombotic syndrome or venous thromboembolic complications. Various factors that contribute to the increasing incidence of DVT in pediatrics have been identified [3]. The increased life expectancy of children with chronic conditions has led to long-term morbidity, which sometimes implies thrombotic complications [4]. Improved diagnostic technologies like ultrasound and CT-scans have increased the accuracy of detecting DVT, while genetic testing has made it easier to identify conditions that make individuals more susceptible to DVT, especially in children with hereditary factors [1]. Furthermore, obesity, a well-established risk for DVT, is prevalent among pediatric patients, leading to an increased risk of blood clot formation by inducing platelet activation, endothelial dysfunction, and an overall inflammatory state [5]. Additionally, sedentary lifestyles with prolonged screen time and reduced physical activity can further contribute to the risk of DVT due to extended periods of immobility [6]. The use of minimally invasive treatments and CVCs in pediatrics has increased, especially in critical patients, leading to a higher risk of DVT due to their thrombotic potential [1]. As a result, the recognition of this disease and the spectrum of associated risk factors are continually advancing, posing a risk of more cases of thrombosis in unexpected patient populations.

**Fig.1:**
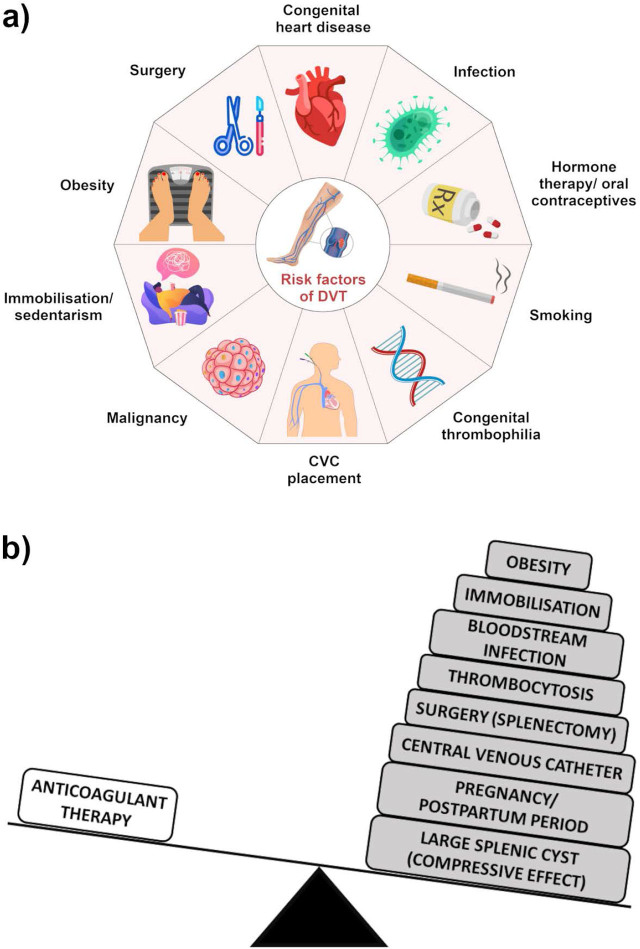
**a) Major risk factors of deep vein thrombosis (DVT). b) Disbalance between the anticoagulant (low-molecular-weight heparin) and procoagulant factors in the pediatric patient.** Image created in GIMP 2.10.30 software (The GIMP Development Team, https://www.gimp.org).

Here, we presented a rare occurrence of DVT in a pediatric patient with multiple simultaneous high-risk factors: obesity, the postpartum recovery period, a large splenic cyst (which increases abdominal pressure), a surgical intervention (splenectomy) leading to secondary thrombocytosis, immobilization during recovery, and the use of a central venous catheter followed by a catheter-related bloodstream infection. Thus, even with the administration of anticoagulant prophylaxis, the patient’s progression was significantly influenced by a plethora of associated risk factors (figure 1b). This underscores the critical need to identify and address such risk factors in clinical practice, emphasizing the limitations of prophylactic treatment in cases with a particularly high-risk profile.

Pediatric DVT presents unique challenges for health specialists, complicating the landscape of diagnosis and management. Unique risk factors (figure 1a) contribute to the complexity of pediatric DVT, leading to the need for heightened clinical suspicion, a personalized prophylaxis strategy, and a careful diagnostic approach [7, 8]. Determining the best preventative strategies for high-risk scenarios is challenging due to a lack of thorough data and established guidelines for the pediatric population [9]. As an example, the variability in the reported outcomes of studies that have investigated the adequacy of treatment and prevention methods for CVC-related thrombotic events underscores the need for a rigorous clinical study to address these critical questions [10, 11]. Clinicians also face numerous challenges when managing DVT during pregnancy and the postpartum period in adolescents, which remains a relatively unexplored territory. As a result, many uncertainties persist for pediatricians and obstetrician-gynecologists alike [12, 13].

There is a noticeable lack of studies and trials that exclusively focus on diagnosing and treating pediatric thrombosis, leading to limited experience and awareness of specific pediatric DVT cases. As emphasized by numerous studies, optimal treatment for pediatric thrombosis is challenging due to physiological differences and therapy impacts across age groups [1, 14]. Anticoagulant therapies and monitoring strategies must be tailored to children’s unique characteristics.

Vitamin K antagonists are less preferred in pediatric populations given their narrow therapeutic window, dietary interactions, and the need for frequent INR monitoring, which is challenging in children [1, 16, 17]. Unfractionated heparin (UFH) can be used in all pediatric age groups, but careful monitoring and dosage adjustments are necessary to maintain its effectiveness while reducing the risk of bleeding. Consequently, UFH is typically reserved for critically ill patients due to its short half-life and the availability of an antidote, protamine sulfate [1, 16, 17]. Low molecular weight heparin (LMWH), such as enoxaparin, is frequently utilized in hospital environments due to its favorable pharmacokinetic profile, prolonged duration of action, and reduced need for monitoring blood parameters. Nevertheless, it has a potential negative impact on bone mineral metabolism, and its effect is only partially reversed by protamine [1, 16, 17]. However, all heparin-based anticoagulant agents can present challenges related to appropriate vascular access or management of subcutaneous injections. Therefore, emerging alternatives such as direct oral anticoagulants (DOAC) offer advantages like oral administration, weight-based dosage, wide therapeutic range, and fewer interactions, but their use requires careful consideration due to limited evidence-based studies and potential challenges in administration and monitoring [15]. Rivaroxaban and dabigatran etexilate are the first two DOACs approved for use in pediatric patients currently. The risks of recurrent clotting and DVT complications must be weighed against the long-term effects of anticoagulant medication, which are difficult to manage and understand in the long run, particularly given the child’s developing and dynamic physiology [16, 17].

Finally, considering psychosocial factors is crucial in pediatrics. The understanding of patients and families and their involvement in managing the disease greatly affect compliance and follow-up [18]. Research indicates that patients with DVT may experience mental health issues like anxiety, depression, and PTSD post-event [19]. Thus, it’s vital to assess and address these factors to optimize treatment outcomes [20].

## Conclusion

As our society undergoes constant changes, the risk factors associated with deep vein thrombosis could also evolve and become progressively more varied, leading to an increase in complex cases, which requires careful management and consideration.

The dynamic nature of pediatric medicine calls for ongoing reassessment of diagnostic approaches and the development of multidisciplinary and individualized therapy strategies tailored to each child’s specific needs.
